# Theoretical and Experimental Study of Radial Velocity Generation for Extending Bandwidth of Magnetohydrodynamic Angular Rate Sensor at Low Frequency

**DOI:** 10.3390/s151229869

**Published:** 2015-12-15

**Authors:** Yue Ji, Xingfei Li, Tengfei Wu, Cheng Chen

**Affiliations:** 1 State Key Laboratory of Precision Measuring Technology and Instruments, Tianjin University, Tianjin 300072, China; jiyue@tju.edu.cn (Y.J.); wtf@tju.edu.cn (T.W.); 2 School of Mechanical Engineering, Tianjin University of Commerce, Tianjin 300134, China; chencheng@tju.edu.cn

**Keywords:** angular rate sensor, magnetohydrodynamic, low frequency expansion

## Abstract

The magnetohydrodynamics angular rate sensor (MHD ARS) has received much attention for its ultra-low noise in ultra-broad bandwidth and its impact resistance in harsh environments; however, its poor performance at low frequency hinders its work in long time duration. The paper presents a modified MHD ARS combining Coriolis with MHD effect to extend the measurement scope throughout the whole bandwidth, in which an appropriate radial flow velocity should be provided to satisfy simplified model of the modified MHD ARS. A method that can generate radial velocity by an MHD pump in MHD ARS is proposed. A device is designed to study the radial flow velocity generated by the MHD pump. The influence of structure and physical parameters are studied by numerical simulation and experiment of the device. The analytic expression of the velocity generated by the energized current drawn from simulation and experiment are consistent, which demonstrates the effectiveness of the method generating radial velocity. The study can be applied to generate and control radial velocity in modified MHD ARS, which is essential for the two effects combination throughout the whole bandwidth.

## 1. Introduction

The angular rate measurement with the stringent requirement in terms of bias drift and resolution has been advanced by development of gyroscopes technologies [1] with outstanding performance like Control Moment Gyros [2], Ring Laser Gyros [3], Fiber Optic Gyros [4] and Hemispherical Resonator Gyros [5]. These gyroscopes cannot be extensively used in harsh environments where vibration and temperature transients are severe [6], even if weight, size, and power consumption could be accepted. Some emerging gyroscopes (e.g., Micro-electromechanical Systems (MEMS) gyros [7] and micro integrated optic gyros [8]) have been developed for those applications assuring miniaturized and low-cost gyroscopes such as in the aerospace industry and consumer electronics markets. While for MEMS gyroscopes the reliability and shock resistance issues [9] are still open and micro integrated optic gyros have serious critical aspect in reducing the influence of noise sources especially in a wide bandwidth [10], in fact, there are some applications where very small angles in vibration environments need to be measured and controlled precisely. On-orbit jitter measurement in line-of-sight (LOS) stabilization system [11], space borne Earth observation [12] and Solar Dynamic Observatory [13] are examples of such environments where ultralow measurement noise (<1 μrad/s(rms)) [14] with wide bandwidth [15] is demanded. The magnetohydrodynamic (MHD) angular rate sensor (ARS) has no mechanical saturation allowing rapid recovery from large angle slews and low measurement noise levels without additional increased complexity in ultra-wideband. Besides, it has low power consumption and cost, and high shock resistance. The MHD ARS has generated considerable recent research interest since the previous patents [16,17,18,19] and been applied for making accurate measurement in harsh environments, such as inertial motion of head kinematics test [20], high-g sled [21] and other automotive safety research [22]. However, the MHD ARS does not possess the capability of measurement at low frequency (<1 Hz) especially constant inertial angular rate [23]. This limitation implies large drift rates and makes the MHD ARS unsuitable for determining angular position over long periods of time [24]. The angular displacement discrepancy calculated by MHD ARS is generated when rotating at low frequency [25]. For applications requiring an inertial pointing reference, a separate source must be utilized for low frequency rate measurement for compensation [26]. In order to break through the limitations of MHD ARS in applications, the low frequency expansion study needs to be studied.

Research has been accelerated in extending the measurement scope of MHD ARS throughout the whole bandwidth. Laughlin *et al.* [27] proposed a “blending filter” to combine high frequency MHD sensor measurement and low frequency rate provided by a conventional gyroscope and then made experiments to prove that the poor performance can be attributed to the weakened relative angular rate between the inertial-fixed fluid and case-fixed magnetic flux at low frequency [28]. Pinney *et al.* [29] presented the spectral description of the noise in the measurement to predict the sensor’s drift performance. Merkle *et al.* [30] made a digital compensation processing to extend the sensor range to lower frequencies. Xu *et al.* [31] studied the theoretical model of MHD ARS by a numerical method and showed that the viscidity and electromagnetic force result in the poor performance at low frequency. It is difficult to break through the inherent performance limitation only by the compensation in dynamic environment [30,32]. Therefore, it is imperative to study a modified physical construction of MHD ARS to sense low frequency rate without affecting its characteristic at high frequency. This paper introduces a radial rate to induce the relative circumferential velocity due to Coriolis acceleration at low rotating frequency, whereas the MHD effect dominates at high frequency. A radial velocity generation by an MHD pump in MHD ARS is proposed and studied by a designed device.

The paper is organized as follows: Section 2 presents the simplified model of the modified MHD ARS combing the Coriolis and MHD effect, in which the importance of radial flow rate is illustrated. Section 3 describes a device generating the radial velocity induced by an MHD pump, whose generated flow rate can be measured. In Section 4, simulation is made to study the design of structure and physical parameters for controlling the radial rate. Experiments with the designed device are shown to verify the validity of the simulation in Section 5. Section 6 summarizes the whole paper.

## 2. Simplified Model of Modified MHD ARS

This section is to describe the measurement principle of the modified MHD ARS introducing Coriolis effect at low frequency and then deduce the simplified model to present the sensor measurement through the whole bandwidth in theory.

### 2.1. Description of the Modified MHD ARS Introducing Coriolis Effect

**Figure 1:**
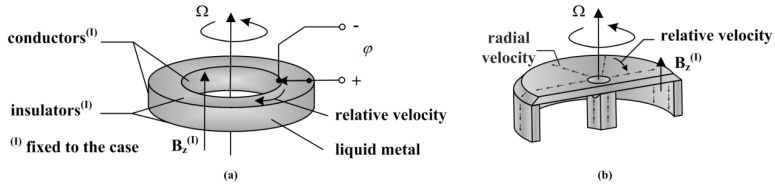
(**a**) Description of the basic MHD ARS; (**b**) Description of the modified MHD ARS.

Figure 1a shows the annual channel of a basic ARS based on MHD effect. As the case rotates with angular velocity Ω, the case-fixed magnetic flux B_z_ moves through the inertial-fixed conducting fluid with relative velocity. The phenomenon that the fluid cuts the magnetic induction lines occurs. Then, the motional electromotive force *Æ* emerges therefrom, which is proportional to the angular velocity Ω.

In Figure 1b, radial velocity is introduced in addition to the relative circumferential velocity between the conducting fluid and rotating case. At low rotating frequency, relative circumferential velocity is weakened by the viscous and electromagnetic force but accelerated due to Coriolis acceleration similar to other Coriolis vibratory gyroscopes [33,34] and then a radially-oriented electric field is generated, whereas the MHD effects illustrated in the basic MHD ARS dominate at high frequency. The measurement through the whole bandwidth is achieved by combing the Coriolis effect at low frequency and MHD effect at high frequency. The scale factor of the two effects must be consistent. Therefore, the key is the generation and control of the radial velocity.

### 2.2. The Simplified Model of the Modified MHD ARS Introducing Coriolis Effect

**Figure 2:**
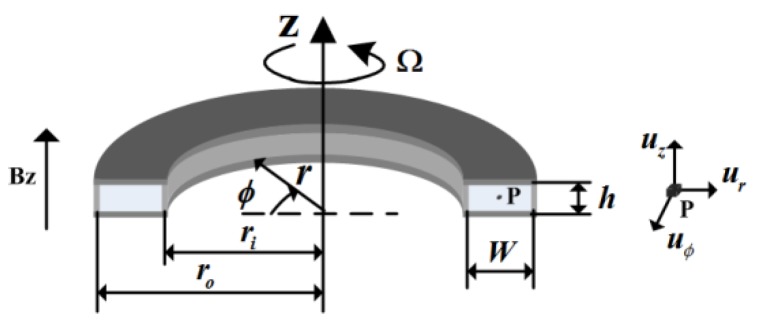
The parameter and coordinates addressed in the section of the annual channel in MHD ARS.

Figure 2 shows the schematic of parameters and coordinates addressed in the section of the annual channel in the modified MHD ARS shown in Figure 1b. In the process of derivation, we use cylindrical coordinates (*φ*, *r*, *z*), where (*φ*, *r*, *z*) are the circumferential, radial and axial directions, respectively, with the axis of curvature defined as *r* = 0 and the middle of the duct in the axial direction defined as *z* = 0. Top and bottom sides are the parallel insulating plate of height *h*; inside and outside walls are concentric conducting cylinders of radii *r_i_* and *r_0_*; and the magnetic field imposed is *B_z_*. The case is rotating at the angular velocity Ω, including the walls of flow and the magnetic flux. The relative velocity in the moving referee frame is expressed in terms of cylindrical components (*u_φ_*, *u_r_*, *u_z_*).

The duct is completely axisymmetric about axis *z*, so *∂P/∂φ*, *∂u/∂φ* are all zero. The fluid motion is laminar and subjected to a body force *f_φ_*, which is Lorentz force. In the moving reference frames of the rotating case, the relative circumferential velocity formulation of Navier-Stokes equations takes the form in the non-inertial system along the circumferential direction:

(1) $$\frac{\partial u_{\phi }}{\partial t}+u_{r}\frac{\partial u_{\phi }}{\partial r}+u_{z}\frac{\partial u_{\phi }}{\partial z}+\frac{u_{r}u_{\phi }}{r}=f_{\phi }-\frac{\partial \Omega }{\partial t}\cdot r-2\cdot \Omega \cdot u_{r}+\nu \left(\frac{\partial^{2}u_{\phi }}{\partial r^{2}}+\frac{1}{r}\frac{\partial u_{\phi }}{\partial r}-\frac{u_{\phi }}{r^{2}}\right)+\nu \frac{\partial^{2}u_{\phi }}{\partial z^{2}}$$

here $\left(u_{r}\partial u_{\phi }/\partial r+u_{r}u_{\phi }/r\right)\sim Ο\left(U_{r}U_{\phi }/R\right)$, $\left(u_{z}\partial u_{\phi }/\partial z\right)\sim Ο\left(U_{z}U_{\phi }/h\right)$, $\nu \partial^{2}u_{\phi }/\partial z^{2}\sim Ο\left({\nu U}_{z}/h^{2}\right)$, $\nu \left(\partial^{2}u_{\phi }/\partial r^{2}+1/r\cdot \partial u_{\phi }/\partial r-u_{\phi }/r^{2}\right)\sim Ο\left({\nu U}_{\phi }/R^{2}\right)$, and ~O means the two equations in the same order of magnitude. *ν* is viscosity coefficient of the fluid. The equivalent radius $R=\left(r_{o}+r_{i}\right)/2$ is defined.

Simplifying the model, the assumptions are made as follows:

Condition 1: For *ϕ* is the main flow direction, $U_{z}\ll U_{\phi }$ is assumed in the simplified model.

For the dimensions of the ARS is in mm and the angular velocity Ω is not too high in applications, the expression $\left(U_{z}U_{\phi }/R\right)/\left({\nu U}_{\phi }/R^{2}\right)=\left(U_{\phi }R/\nu \right)\cdot \left(U_{r}/U_{\phi }\right)\sim Ο\left(\left({\Omega R}^{2}/\nu \right)\cdot \left(U_{r}/U_{\phi }\right)\right)<Ο\left(1\right)$ can be given. In general, the height *h* is smaller than the equivalent radius *R*. Therefore, ${\nu U}_{\phi }/R^{2}$ is much smaller than ${\nu U}_{\phi }/h^{2}$. As a result, $U_{z}U_{\phi }/R$ and ${\nu U}_{\phi }/R^{2}$ can be ignored compared with ${\nu U}_{\phi }/h^{2}$. Besides, for $U_{\phi }/R\leq \Omega$, we can get the expression $U_{r}U_{\phi }/R\leq 2\cdot \Omega \cdot U_{r}$. The Equation (1) can be written as:

(2) $$\frac{\partial u_{\phi }}{\partial t}=f_{\phi }-\frac{\partial \Omega }{\partial t}\cdot r+\nu \frac{\partial^{2}u_{\phi }}{\partial z^{2}}-2\cdot \Omega \cdot u_{r}$$

Then, we can consider the flow as 2D Poiseuille flow between the top and bottom sides, taking the height *h* as characteristic length. The steady-state solution of the fully developed velocity can be presented as $V=-(1/2\nu \rho )\cdot \left(\partial p/\partial z\right)\cdot \left(h^{2}-z^{2}\right)$. Considering the conditions $V_{max}=-(1/2\nu \rho )\cdot \left(\partial p/\partial z\right)\cdot h^{2}$. and $V_{max}=u_{\phi }$, we can obtain the expression $\partial^{2}u_{\phi }/\partial z^{2}\sim Ο\left(\left(-1/h^{2}\right)\cdot u_{\phi }\right)$. Based on the assumptions above, Equation (2) can be written as:

(3) $$\frac{\partial u_{\phi }}{\partial t}=f_{\phi }-\frac{\partial \Omega }{\partial t}\cdot r-2\cdot \Omega \cdot u_{r}-\nu \cdot u_{\phi }/h^{2}$$

Condition 2: Only taking the current induced by *u_ϕ_* into consideration and ignoring the induced magnetic fields under the assumption of small Reynolds number, $f_{\phi }=j_{r}\times B_{z}$ can be reached. According to the Ohm’s law and the Maxwell equation, we have the equation $f_{\phi }=1/\left({\rho \sigma B}_{0}^{2}u_{\phi }\right)$, in which *σ* and *ρ* are respectively electrical conductivity and mass density. For the Lorenz force opposing the relative motion, *f_ϕ_* is negative.

The Laplace transform of Equation (3) is:

(4) $${su}_{\phi }+{\sigma B}^{2}u_{\phi }/\rho +\nu \cdot u_{\phi }/h^{2}=-s\Omega \cdot r-2\cdot \Omega \cdot u_{r}$$

According to the law of electromagnetic induction, a voltage difference along the radial direction *φ* can be calculated.

(5) $$\left|\frac{\varphi \left(s\right)}{\Omega \left(s\right)}\right|=\int_{r_{i}}^{r_{o}}\left(B_{z}u_{\phi }\right)\cdot dr=\frac{B_{z}W\left(R\cdot s+2u_{r}\right)}{s+\sigma B^{2}/\rho +v/h^{2}}=\frac{B_{z}WR\left(s+2u_{r}/R\right)}{s+v/h^{2}\left(1+{Ha}^{2}\right)}$$

**Figure 3:**
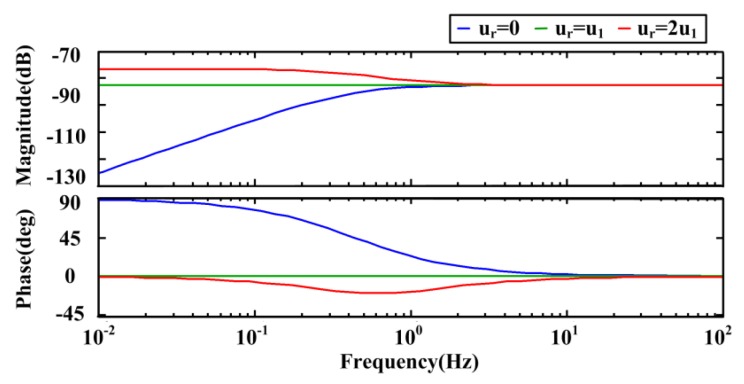
Frequency response curves of the simplified model at different radial velocity.

Here, *Ha* is the Hartmann number ($Ha=\sqrt{{\sigma B}_{z}^{2}h^{2}/\left(\nu \cdot \rho \right)}$). It gives the ratio of Lorentz force and frictional force. From the expression, we can draw the conclusion that the expression $\left|\varphi \left(s\right)/\Omega \left(s\right)\right|=B_{z}WR$ can be reached in the whole passband when the expression $u_{r}=u_{1}=\left(R/2\right)\cdot 2\pi \cdot f_{z}=\left(R/2\right)\cdot \left(\nu \left(1+{Ha}^{2}\right)/h^{2}\right)$ can be satisfied. Otherwise, the transition of amplitude and phase frequency response at the frequency $f_{z}=\nu \left(1+{Ha}^{2}\right)/2\pi h^{2}$ would exist, and then the ratio of the voltage φ and the angular rate Ω would be quite different between low frequency and high frequency, which can be clearly illustrated in Figure 3.

The frequency response curves of the transfer function at *u_r_* = 0, *u_r_* = *u_1_* and *u_r_* = 2*u_1_* are plotted, in which the parameters *f_z_* = 0.45 Hz, *B_z_* = 0.2 T, *W* = 22 mm and *R* = 17 mm are supposed. If the radial velocity is larger than the anticipated value, the amplitude is higher at low frequency and the phase is below zero around the transition frequency. The radial flow velocity directly affects the performance of the sensor. Therefore, the generation and control of the radial rate should be studied to combine the two effects well.

## 3. The Device to Study the Radial Velocity Generation by MHD Pump (DRVG)

A radial velocity generation method by a MHD pump in the central channel is described in the section. However, the radial rate provided in the modified MHD ARS is highly coupled with circumferential velocity and cannot be measured directly in the sensor experiment. Therefore, a device to study the radial velocity generation should be designed. Flow rate is always obtained by measuring the pressure head difference and flow rate in import and export [35,36] or by some flow sensors [37]. However, the conducting fluid is easily oxidized or volatile and the fluid channel in MHD ARS is closed. The evaluation method with measurement in the opening holes is unsuitable for radial velocity measurement in MHD ARS. For the conducting fluid, we choose to take a measurement in the return channel according to electromagnetic induction principle, which will be presented in the following.

### 3.1. Description of the Design

**Figure 4:**
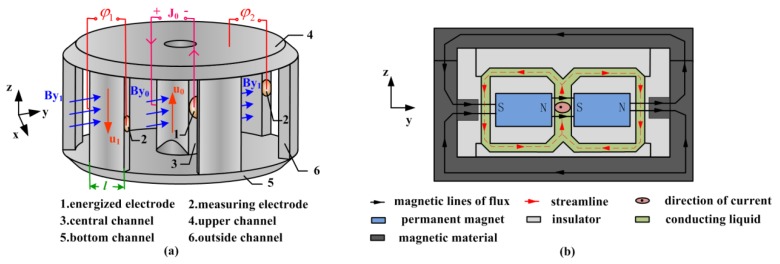
(**a**) Fluid channel of DRVG; (**b**) Sectional view of DRVG in *yz* plane.

Figure 4 presents a device generating the radial rate in the annual channel by an MHD pump, which is abbreviated as DRVG for convenience in following. Figure 4a shows the fluid channel of DRVG. The fluid is forced upward at velocity u_0_ along *z* axis by MHD pump in the central channel, which is constituted of a magnetic field **B_y0_** along the *y* axis and an electric current **J_0_** provided by the two energized electrodes on both sides of the central channel in *x* direction. The fluid is then forced radially outward in the upper channel and travels through the outside channel to the bottom channel. The upper and bottom channel both can act as the annual channel in MHD ARS illustrated in Figure 1b.

As seen in Figure 4a, the return channel is divided into six uniform distribution channels to sense the flow rate. Among them, the two channels perpendicular to the magnetic field in *y* direction are chosen to detect the flow rate and the measuring electrodes are designed to sense the generated voltage. The expression *u_1_* = *φ_1_*/(*B_y1_*⋅*l*) can be approximately obtained, in which *l* is the length of the outside channel.

The sectional view of DRVG in *yz* plane is shown in Figure 4b. Two permanent magnets are placed on both sides of central channel in *y* direction. Magnetic material is used as a shell to form the closed magnetic field lines to ensure that the magnetic field in the central and the two return channels are all in the *y* direction. The conducting fluid is forced by the MHD pump and flows along the streamline as shown.

### 3.2. The Government Equations in DRVG

In the DRVG, the magnetic Reynolds number is assumed small, which allow us to use quasi-static approximation [38,39]. The influence of the flow velocity on the magnetic field is neglected and the flow is described by the Navier-Stoke system with an additional Lorenz force term, Ohm’s law for the density of induced electric currents on incompressibility condition, and the potential equation expressing the constraint of charge conservation [40]. Gravity needs to be overcome in the DRVG, so it should be taken into consideration. The dimensions of the fluid channel in MHD ARS are in the order of mm, so the “slip” [41] is ignored in the present study. The governing equations can be expressed as:

(6) ∇⋅u=0

(7) $$\frac{\partial u}{\partial t}+u\cdot \nabla u=F-\frac{1}{\rho }\nabla p+\nu \nabla^{2}u+\rho \cdot g$$

(8) J=σ(−∇φ+u×B)

(9) $$\nabla^{2}\varphi =\nabla \cdot \left(u\times B\right)$$

(10) F=J×B

Total magnetic field, the electric current density, the pressure field and the relative velocity field are denoted by **B**, **J**, **p** and **u**, respectively.

**Figure 5:**
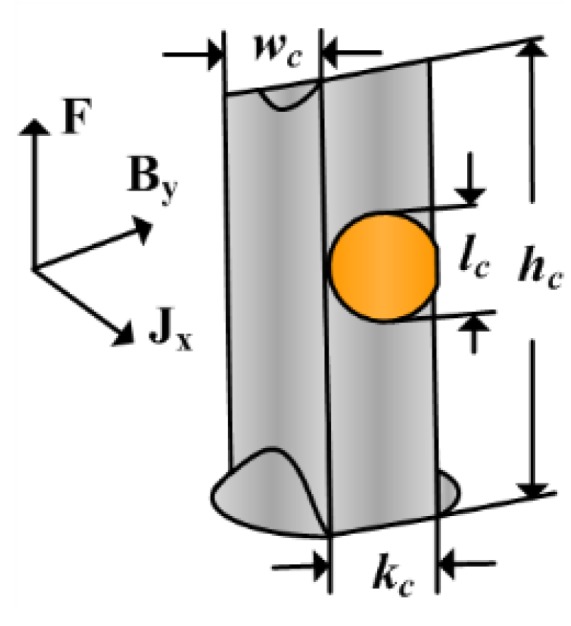
Variables of the pump in the central channel.

Accurate prediction of MHD flow can only be obtained by sophisticated numerical simulation. However, a simple expression for volumetric flow rate may approximately be obtained from the Hagen-Poiseuille equation [42]:

(11) $$u\propto \frac{{iB}_{y}k_{c}w_{c}^{2}}{8{\rho \nu l}_{c}{\left(k_{c}+w_{c}\right)}^{2}}$$

where variables of the pump in the central channel are shown in Figure 5 along with range used for this paper. The section of the energized current is round in the design, so the variable *k_c_* equals to *l_c_*. Therefore, the Equation (11) shows that the flow rate is proportional to current *i*, magnetic field *B_y_* and width *w_c_*, and, inversely, to the thickness *k*_c_ of the channel.

## 4. Simulation of DRVG

The numerical simulation is performed using the finite volume computational fluid dynamics software package ANSYS FLUENT coupled with the electromagnetic field. The mechanical structures and physical parameters are all listed in Table 1. The parameters of annual channel mostly depend on the demand of ARS, which are not analyzed in this paper. The width of the pump *w_c_* should be equal to the inner radii of upper and bottom channel *r_i_*. The variables marked with * are pivotal for generating radial velocity, which is studied in simulation. The faces except the electrodes are all non-conducting, whereas the energized and measuring electrodes are conducting materials to apply the electric current and acquire the voltage. The energized current is controlled by the current density J on the face of energized electrodes. The direction of the current is set as shown in Figure 4a to make the fluid flow upwards in the central channel. When driven at every current in the MHD pump, the velocity in the central channel and the two outside channels approximatively in *x* direction are recorded. Besides, the potential difference between the energized and measuring electrodes is observed.

**Table 1:** Simulation parameters of the device to study the radial velocity generation (DRVG).

Mechanical Structure Parameters		Physical Parameters of Fluid	Mercury
Inner radius of annular channel *r_i_*	6	Density *ρ* (kg/m^3^)	1.354 × 10^4^
Outer radius of annular channel *r_o_*	28	Electrical conductivity *σ* (1/Ω⋅m)	1.044 × 10^6^
Height of upper and bottom channel *h*	3	Kinematic viscosity *ν* (m^2^/s)	1.125 × 10^−7^
The length of every channel *l*	11.65	Magnetic permeability *μ* (H/m)	1.257 × 10^−6^
Radian of every outside channel (deg)	24	Facet of energized and electrodes electrodes	conducting
Thickness of outside channel	3	The surface except electrodes	insulating
Diameter of measuring electrode	2	Gravity	√
Thickness of central channel *k_c_*	*	Magnetic field intensity in central channel B_y0_(T)	0.6
Area of energized electrodes S_e_ = π(*k_c_*/2)^2^		Magnetic field intensity in outside channel B_y1_(T)	0.5
Height of central and outside channel *h_c_*	*	Energized current density J	*

### 4.1. Influence of Central Channel’s Thickness k_c_

**Figure 6:**
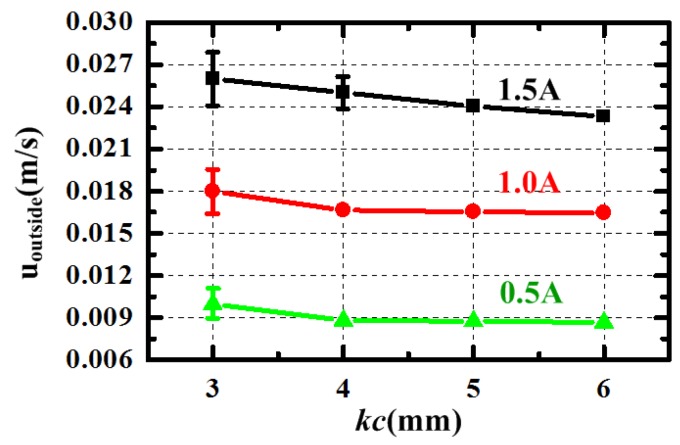
The generated velocity *u_outside_* changing with different central channel’s thickness *k_c_* at energized current 0.5, 1.0 and 1.5 A.

From the Equation (11), we can know that the generated velocity *u_outside_* becomes smaller as the thickness of central channel *k_c_* is larger, which is also displayed in Figure 6. It is evident that the generated velocity pumped by the same current at *h* = 3 mm is the most large. However, its fluctuation exists at every current and becomes bigger as the current increases. When the thickness is 3 mm, the MHD pump cannot make the velocity in the closed fluid channel steady. Besides, the velocity in the model at *h* = 4 mm also becomes unsteady when the energized current is 1.5 A. To explore the performance of the device at high current, the thickness *k_c_* in the design is set at a larger value 6 mm. The simulation result illustrates that the thickness *k_c_* has negative correlation with generated velocity; however, the velocity cannot become steady when the thickness is too small especially at high-energized current.

### 4.2. Influence of Central and Outside Channel’s Height h_c_

**Figure 7:**
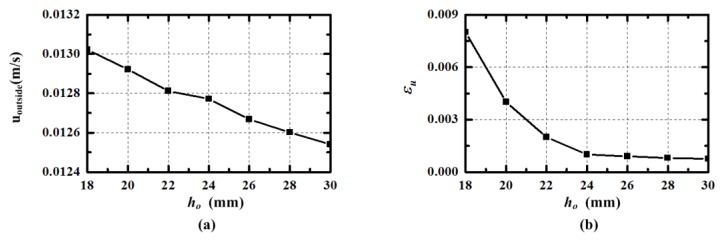
The velocity *u_outside_* and its error *ε_u_* in outside channel pumped by *J* = 25,000 A/m^2^ (*I* = 0.66 A) varying with the central and outside channel’s height.

The performance of the models with different height *h_c_* are simulated and the generated velocity *u_outside_* pumped by *J* = 25,000 A/m^2^ (*I* = 0.66 A) are shown in Figure 7a. The volume of the fluid needed to pump increases as the height *h_c_* is larger. As expected, the velocity *u_outside_* has negative correlation with the height *h_c_*. However, the volume of the sensor is proportional to the height *h_c_*. Besides, the decreasing amount of flow velocity from *h_c_* = 18 mm to *h_c_* = 30 mm is only about 3.85%, which is not significant. The error rate *ε_u_* of the developed radial velocity is recorded, which is calculated from the results in 1000 intervals after the velocity becomes steady. The error rate *ε_u_* of different models are given in Figure 7b, and it decreases when the height *h_c_* increases. When the height *h_c_* is larger than 24 mm, the slope becomes small and it seems likely that the velocity in the central channel can be developed. Considering the generated velocity *u_outside_* and its error, the suitable value of the height is 24 mm in the design. The simulation results indicate that the height *h_c_* should be designed large enough to make radial rate error acceptable, under the premise that the volume is allowable.

### 4.3. Influence of Energized Current Density J

**Figure 8:**
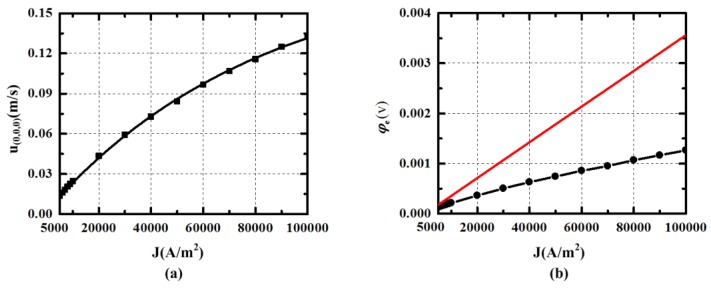
The velocity *u*(0,0,0) in central channel and the potential difference *φ_e_* between the two energized electrodes generated by the pump at current density J in simulation.

According to the analysis in Section 4.1 and Section 4.2, the parameters *k_c_* and *h_c_* are respectively set at 6 mm and 24 mm. In simulation, the velocity cannot be developed, when the energized current density *J* is smaller than 5000 A/m^2^. It means that the current should be larger than 0.1 A in order to make the pump running in the device. In Figure 8a, the velocity *u*_(0,0,0)_ in the middle of the central channel generated by the energized current density *J* is shown. The fitting curve is plotted and can be expressed as the equation *Y(u_(0,0,0)_)* = −0.18378⋅*exp(−x(J)/82452)* + 0.18623. From the expression, it can be seen that the incremental of the velocity is decreased as the current is increased. This can attribute to the increasing viscous force which is proportional to the velocity. The relationship of the current density J and the potential difference *φ_e_* between the two energized electrodes is presented in Figure 8b. The tangent line at J = 5000 A/m^2^ is plotted to make comparison, and it can be found that the impedance of the fluid is decreased as the velocity becomes larger. On the other hand, the ratio of the potential difference and the current can be used as a parameter to characterize the pumped velocity.

**Figure 9:**
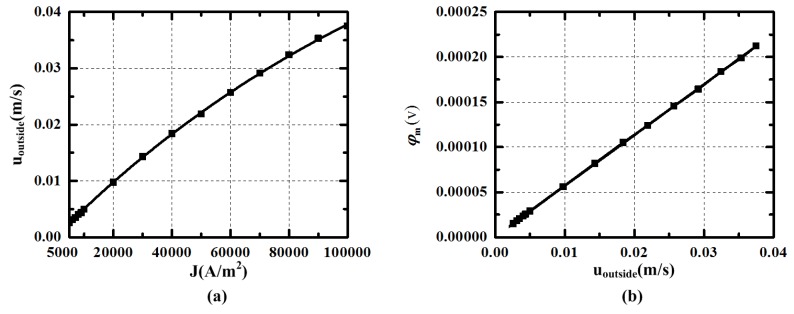
(**a**) The velocity *u_outside_* in outside channel varying with current density J (*u_outside_* is the facet average velocity at r = 28 in the outside channel); (**b**) The potential difference *φ_m_* between measuring electrodes varying with current density J in simulation.

The velocity in upper, bottom and outside channel may be declined by viscous force. The relationship of the velocity *uoutside* and the current density *J* is shown in Figure 9a. The fitting expression can be written as Equation (12), whose goodness of fitting is 0.99986. The equation can be used to calculate the value of the current needed for generating radial rate in modified MHD ARS. For example, if the radial velocity in the bottom channel needs to be set at 1 × 10^−2^ m/s, the current density *J* should be about 20467 A/m^2^. Figure 9b presents the voltage between the measuring electrodes generated by the velocity *uoutside* in the magnetic field By1. The relationship is almost linear and meets the expression *φ_m_* = *B_y1_*⋅*u_outside_*⋅*l*. It shows that the velocity in the outside channel can be measured by the voltage between the measuring electrodes.

(12) $$Y\left(u_{outside}\right)=-0.07563\cdot exp\left(-x\left(J\right)/144161\right)+0.07562$$

## 5. Experiment of DRVG

**Figure 10:**
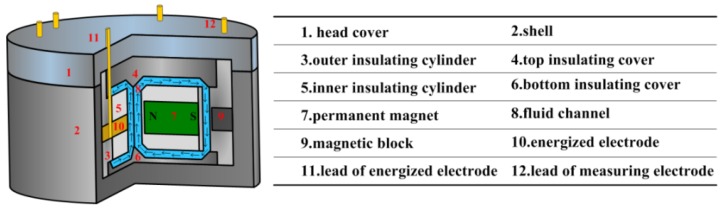
The designed device of DRVG in experiment.

In order to verify the effectiveness of the generation and control of the radial rate by MHD pump in the MHD ARS, a device shown in Figure 10 is devised to make experiment at different energized current. Its parameters of fluid channel are as the same as in the simulation listed in Table 1, in which the variables *l_c_*, *h_c_* are respectively 6 mm, 18 mm. The size of the DRVG prototype is 70 mm × 40 mm × 40 mm and the mass is about 600 g, which is designed for experiment and can be miniaturized in application. The conducting fluid container is formed by the top and bottom insulating cover 4,6 with inner and outer insulating cylinder 3,5. The closed magnetic circuit composes of the shell 2 and the head cover 1 threaded connection with high permeability material, thereby avoiding the interference of external stray electromagnetic field. Besides, the magnetic block 9 is designed to strengthen the magnetic field intensity in the two measuring outer channels. The MHD pump in the central channel is constituted of the magnetic field generated by two permanent magnets 7 and the perpendicular electric current provided by the two energized electrodes 10. The measuring electrodes lies on both sides of the two outside channels faced the magnet to make sense the flow rate. The external power supply is applied by the lead of energized electrodes 11. The lead of measuring electrodes 12 are used to connect the millimeter to sense the generated voltage. 

**Figure 11:**
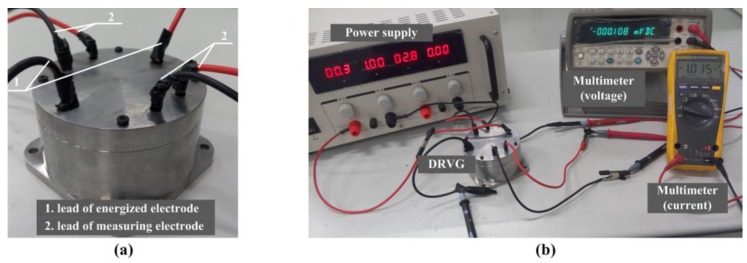
(**a**) Physical map of the DRVG device; (**b**) Physical map of the experimental procedure.

The physical map of the DRVG device and the experimental procedure are shown in Figure 11. The power supply is used to apply the current, which is measured by the $3\frac{1}{2}$ digital multimeter in series connection of circuit. The direction of the current is set as the same as in the simulation to pump the fluid in the central channel upwards. The output of the voltage between the measuring electrodes is measured by a $6\frac{1}{2}$ digital multimeter 34,410 A.

**Figure 12:**
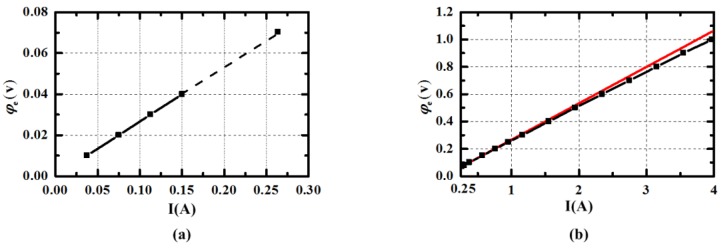
The voltage *φ_m_* between the energized electrodes when applied different current *I*. (The dotted portion means that the stable display cannot be acquired).

**Figure 13:**
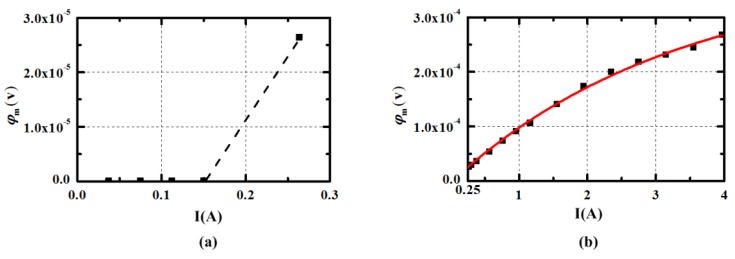
The voltage *φ_m_* between the measuring electrodes when applied different current *I*. (The dotted portion means that the stable display cannot be acquired).

The relationship of the energized current *I* and the voltage *φ_e_* between the energized electrodes is given in Figure 12. Figure 13 illustrates the voltage *φ_m_* between the measuring electrodes when applied different current. When the energized current is lower than 0.12 A as indicated in Figure 12a, the ratio of the voltage *φ_e_* and the current *I* is a constant value 0.2667 Ω, which is much larger than in the simulation. The increasing impedance may be linked with the resistance of the wire. It can be seen in Figure 13a that the voltage *φ_m_* is almost zero in the range of energized current. It is likely that the fluid cannot be pumped in the small current for viscous force and gravity. As the current is increased to 0.28 A, the display of the current and measuring voltage cannot both be stable to be acquired. This may be caused by the critical state of the pump in practical experiment. As presented in Figure 12b and Figure 13b, the incremental of the voltage *φ_e_* and *φ_m_* becomes smaller as the energized current is increased, when the current is higher than 0.28 A. The phenomenon confirms the decreasing resistance caused by the increasing flow rate. The red tangent line at *I* = 0.28 A in Figure 12b is plotted to make comparison to show the change of the resistance clearly. The fitting curve of the voltage *φ_m_* and energized current *I* is given in Figure 12b and the expression can be written as Equation (13). The smaller deviation may owe to the measurement error of digital multimeter and can be acceptable. When the *uoutside* needs to be set at 1 × 10^−2^ m/s the voltage *φ_m_* is 5.825 × 10^−5^ V calculated by the expression *φ_m_* = *B_y1_*⋅*u_outside_*⋅*l*. According to the Equation (13), the calculation of energized current *I* should be set at 0.571 A and the corresponding current density J is 20,195 A/m^2^, which is nearly equal to the simulation results. The power consumption is about 0.09 W when *I* = 0.571 A.

(13) $$Y\left(\varphi_{m}\right)=-3.88073e^{-4}\cdot exp\left(-x\left(I\right)/3.34695\right)+3.85448e^{-4}$$

## 6. Conclusions

The problem of controlling the radial flow rate generated by the MHD pump in the MHD ARS has been studied. It can be used to induce the Coriolis acceleration to improve the sensor’s performance at low frequency. We have deduced the simplified model of the modified MHD ARS to show the combination of the Coriolis and MHD effect to sense angular velocity through the whole bandwidth, whose performance mostly depends on the generation of the radial velocity. Therefore, the study of radial flow rate induced by the energized current in a vertical magnetic field is necessitated. A device was designed to study the radial velocity generation in the MHD ARS, in which the velocity in return channel can be measured to evaluate its performance. The influence of the important parameters on generating radial velocity have been simulated and analyzed. We can draw the conclusions on the design: (1) the thickness of the central channel has negative correlation with generated velocity, however the velocity cannot become steady when the thickness is too small; (2) The central and outside channel’s height should be designed large enough to make radial rate error acceptable, under the premise that the volume is allowable; (3) The incremental of the radial velocity becomes smaller as the current is increased and the current needed to be energized can be calculated by the analytical expression drawn from simulation results. Furthermore, the experiment of the designed device was devised. The feasibility of the method generating radial velocity is demonstrated and the experiment results compare well with the simulation.

The study offers an understanding of the method generating radial velocity in the modified MHD ARS to improve its performance at low frequency, which is essential for the two effects combination. In the further study, the modified MHD ARS is expected to expand the bandwidth from 1–1000 Hz to 0–1000 Hz without disturbing the sensitivity and noise floor [29] 1 μrad/s (rms) achieved by MHD effect at high frequency. Besides, the target of bias drift in the developing modified MHD ARS is supposed at the order of 1 °/h or less. The error source analysis in combination should be explored in future work.
